# Aberrant Expression of microRNA Clusters in Head and Neck Cancer Development and Progression: Current and Future Translational Impacts

**DOI:** 10.3390/ph14030194

**Published:** 2021-02-27

**Authors:** Li-Jie Li, Wei-Min Chang, Michael Hsiao

**Affiliations:** 1 Genomics Research Center, Academia Sinica, Taipei 115, Taiwan; xu3xu4ru56lily@gmail.com; 2 School of Oral Hygiene, College of Oral Medicine, Taipei Medical University, Taipei 110, Taiwan; weiminchang@tmu.edu.tw; 3 Department of Biochemistry, College of Medicine, Kaohsiung Medical University, Kaohsiung 807, Taiwan

**Keywords:** microRNA cluster, translational application, HNSCC

## Abstract

MicroRNAs are small non-coding RNAs known to negative regulate endogenous genes. Some microRNAs have high sequence conservation and localize as clusters in the genome. Their coordination is regulated by simple genetic and epigenetic events mechanism. In cells, single microRNAs can regulate multiple genes and microRNA clusters contain multiple microRNAs. MicroRNAs can be differentially expressed and act as oncogenic or tumor suppressor microRNAs, which are based on the roles of microRNA-regulated genes. It is vital to understand their effects, regulation, and various biological functions under both normal and disease conditions. Head and neck squamous cell carcinomas are some of the leading causes of cancer-related deaths worldwide and are regulated by many factors, including the dysregulation of microRNAs and their clusters. In disease stages, microRNA clusters can potentially control every field of oncogenic function, including growth, proliferation, apoptosis, migration, and intercellular commutation. Furthermore, microRNA clusters are regulated by genetic mutations or translocations, transcription factors, and epigenetic modifications. Additionally, microRNA clusters harbor the potential to act therapeutically against cancer in the future. Here, we review recent advances in microRNA cluster research, especially relative to head and neck cancers, and discuss their regulation and biological functions under pathological conditions as well as translational applications.

## 1. microRNA Biogenesis

**Figure 1:**
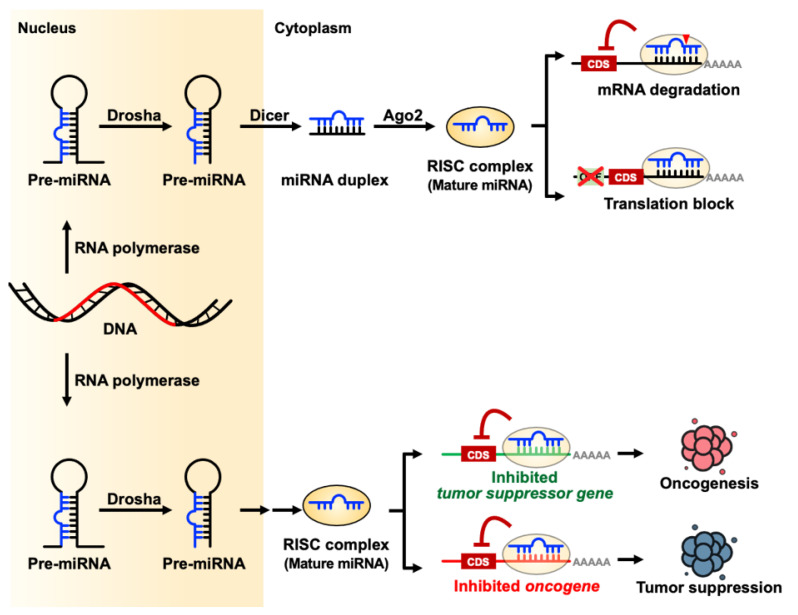
microRNA biogenesis and their biological functions.

Small non-coding RNAs (sncRNAs) are single strand RNA usually < 200 nucleotides in length and include but are not limited to microRNAs, small nucleolar RNAs (snoRNAs), small nuclear RNAs (snRNAs), Piwi-interacting RNAs (piRNAs), endogenous short interfering RNAs (siRNAs), small rDNA-derived RNAs (srRNAs), ribosomal RNA derived fragments (rRFs), transfer RNAs (tRNAs), and their derived small RNAs (tsRNAs) by their origin, length, and functions [1,2,3,4,5,6]. Most sncRNAs are transcripted by RNA polymerase II. Both snoRNAs and snRNAs are larger sncRNAs which length are between 60–140 nucleotides and about 150 nucleotides, respectively. SnoRNAs contribute to RNA modification and snRNA are components in spliceosome. TsRNAs and srRNAs are by-product from tRNA or rRNA and have diverse functions in cell biology. PiRNAs and microRNAs have similar manner to protein coding genes but have different size and interaction proteins. Usually, piRNAs are 26–31 nucleotides in length which interact with piwi-subfamily Argonaute protein and serve as RNA-mediated adaptive immunity against transposon expansions and invasions [7]. MicroRNAs are created by Drosha and Dicer endoribonuclease as 21–24 nucleotides length, that can act as endogenous negative gene regulators in the cells via translational inhibition or mRNA degradation [8]. MicroRNAs have both canonical (Drosha-Dicer and DGCR8 dependent) and noncanonical (Drosha-DGCR8 and Dicer independent but TUTase dependent) pathways to produce mature-form, single-strand microRNAs [9]. The mature-form microRNA enters an RNA-induced silencing complex (RISC) and act as a guidance RNA to regulate the expression of target genes by seed region sequence. RISC-microRNA complex can use microRNA seed region to target microRNA binding elements (MREs) target gene’s 3′-untranslation region (3′-UTR). MicroRNAs act by fine-tuning gene expression through a post-transcriptional mechanism. Due to the multiple-target regulation functions of microRNAs, microRNAs have critical roles in diverse biological functions such as embryogenesis, development, and physiological processes [10] (Figure 1).

## 2. microRNA Clusters

Evolutionary conservation is not only observed in microRNA–target interactions in diverse species [11] but also in genetic order, forming specific microRNA clusters during genomic organization [12]. Approximately 25% of human and 50% of Drosophila melanogaster microRNAs are clustered on chromosomes. MicroRNA clusters contain at least two or more microRNA genes, which are chromosomal adjacent sequences in the same orientation that are not separated by transcriptional units and may be encoded in opposite directions. Usually, cluster microRNAs are also belonged to the microRNA families with the same seed region sequence which may target the same set of genes and may functional redundancy and cooperation among different miRNAs [13,14] Cluster microRNAs are transcriptionally regulated by the same transcriptional factors or single cellular signaling pathways which can be regulated by single and simple activator or repressor protein. Cluster microRNAs are transcribed into large single RNA transcript which are rapidly spliced and processed by the Drosha-DGCR8 complex into individual primary microRNAs [15,16]. Then Dicer and RISC guide mature microRNA on their target genes.

According to miRBase (Version 22), the human genome has 1971 precursor microRNAs [17]. Among the human microRNAs, 468 microRNAs belong to 153 microRNA clusters [18]. These microRNA clusters are distributed across different chromosomes, and chromosome X, which has the highest microRNA clusters numbers among human chromosomes. There are 18 microRNA clusters on chromosome X. The largest microRNA cluster is on chromosome 19. It has 46 microRNAs. The second large microRNA cluster is on chromosome 14q32.2, which has 42 microRNAs [19]. Based on the microRNA distribution in the genomic region, microRNAs are categorized into intergenic, intronic, and exonic microRNA in the genome. About half microRNAs are intragenic microRNAs including one or few intronic and exonic microRNAs which embedded in intron or exon region of host gene on the same strand [20]. Intragenic microRNAs may either share the same promoter with host genes or have independent transcription by itself. During microRNA maturation process, the intragenic microRNAs are byproduct of host gene transcription which may compete to splicing nucleotide sequence elements of precursor mRNA or few in protein coding sequence and also protein–mRNA complex such as spliceosome and the microprocessor in mature mRNA formation [21,22]. These competitions may reduce the intragenic microRNA cluster formation. There are 76 intergenic clusters that have more than 65 intronic and exonic clusters in human genome. In contrast to regulation of intragenic microRNA cluster, the intergenic microRNA clusters have exclusive regulation mechanisms and tightly control in embryogenesis [23], signifying that intergenic microRNA clusters have more important roles in the control of gene expression and biological functions [18].

MicroRNA clusters not only have multiple crucial physiological roles under normal conditions related to the maintenance of cell hemostasis, but they also play roles in pathological conditions when they are dysregulated. microRNA clusters are involved in biological functions, including differentiation and development, nervous system regulation, immunity balance, DNA repair, cell junction and adhesion molecule regulation, intercellular commutation or as inner cellular secondary messengers, mammalian or cellular reproduction, metabolism homeostasis, cell reprogramming and the mesenchymal to epithelial transition or vice versa, organellar biogenesis and function, and responses to stressful conditions which we have summarized physiological microRNA cluster function in Table 1.

Specifically, cluster miR-17/92 has multiple roles from basic cell biology to whole physiological systems, such as DNA repair, secondary messenger, embryogenesis, developments, inter-cell commutation and immunity response [24]; cluster miR-23/24 is involved in stress response, acting as secondary messenger metabolism, organellar biogenesis and function, feedback loop, controlling immune response, and cell junction and adhesion [25,26]. Cluster miR-106b/25 is important in differentiation and development, cell reprogramming and as secondary messengers in stress and viral infection response [27]. Cluster miR-302b/367 has roles in differentiation and development, organellar biogenesis and function, metabolism, reproduction, cell reprogramming and mesenchymal to epithelial transition (MET). Cluster miR-439/136 and 379/656 are belonged to same genetic imprinting domain and they have critical function in differentiation, development, and nervous system regulation, metabolism, and cell to cell commutation [19,28]. Cluster miR-424/450b controls differentiation, development, MET, metabolism, and stress response [29]. Cluster miR-34b/34c is important in differentiation, development, stress response and reproduction [30]. Cluster miR-212/132 is important in nervous system development and regulation [31].

Both cluster miR-301b/130b and miR-508/513a are important in DNA repair system [32,33]; cluster miR-1/133a, miR-143/145, miR-15a/16-1 in cardiac development and disease and as secondary messengers; cluster miR-371a/373 in mammalian and cellular reproduction; cluster miR-106b/25 in metabolism and organelle biogenesis and function; cluster miR-512/519 in development and cell commutation. Both cluster miR-181a/181b and miR-183/182 are important in immunity response and they have their unique role in nervous system regulation and metabolism, respectively. Moreover, for the cluster miR-183/182, it is missed in the scheme of miR-96 that is part of the cluster and it is missed their roles in development and differentiation in the visual, auditory, vestibular, and olfactory systems [34,35,36]. The cluster miR-181 family, that is an evolutionary conserved family microRNAs organized in 3 clusters that have several different roles in central nervous system and immune system development, neurodegeneration and cancer [37,38,39]. Cluster miR-200b/429 is involved in feed-back loop regulation; cluster miR-199a/214 and miR-27a/27b contribute to organellar biogenesis and functions; cluster miR-29b/29a is crucial in nervous system regulation; cluster miR-193/365a controls metabolism. Cluster miR-206/133b contributes to differentiation and development and cluster miR-211/222 acts as cellular secondary messengers.

The diversity of microRNA cluster functions was clearly described by Kabekkodu et al. [18]. MicroRNA clusters have huge impacts on cells, especially in neoplasia initiation, tumorigenesis, microenvironment commutations, and metastasis [40].

## 3. Regulation of microRNA Clusters

MicroRNA clusters have important roles in the maintenance of normal cellular hemostasis and commutation to surrounding microenvironments. MicroRNA cluster expression is controlled by multiple mechanisms by genetic or epigenetic events and the microRNAs. Mutation or single nucleotide polymorphisms (SNP) on microRNA cluster promoter region changes the transcription factor binding ability which regulate cluster microRNAs expression [41]. About 50% of microRNAs are located at fragile chromosome sites where the genomic regions suffer from copy-number changes in the mitosis [42]. Cancer cells are quickly dividing cells that easily cause replication stress which can promote accumulation of genetic mutation such as chromosome recombination, or translocation, amplification, deletion, or loss of heterozygosity upon alignments of homologous chromosome.

**Figure 2:**
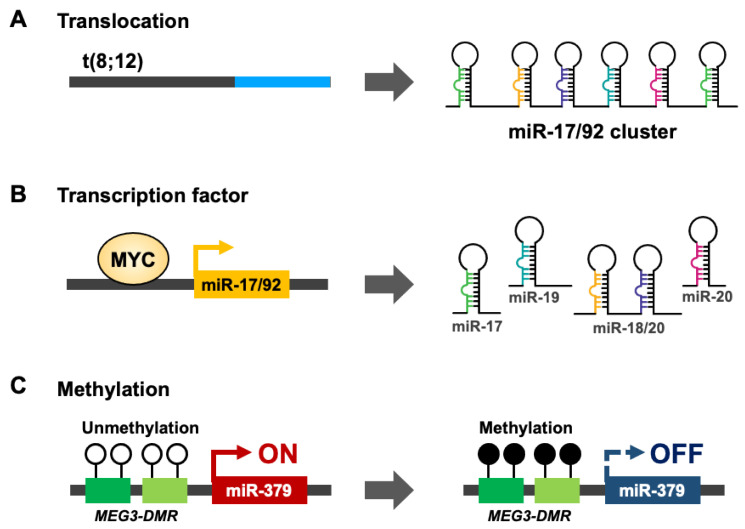
The regulation mechanisms of microRNA clusters in carcinogenesis. (**A**,**B**) Expression of microRNA cluster is regulated by genetic events. miR-17/92 cluster is stimulated by chromosome t(8;12) translation (**A**) or transcriptional activation by transcription factor MYC (**B**). (**C**) The epigenetic regulation of miR-379/656 cluster upon DNA hypermethylation on MEG3-DMR.

HNSCC is not common cancer model for mechanism studies of microRNA cluster regulation. Thus, we summary the potential mechanisms from other cancer types. In ovarian cancer [43,44,45], Burkitt’s lymphoma [46], and diffuse large B-cell lymphoma, miR-200b/429 [47] or the miR-17/92 cluster [24] undergo copy number amplification to stimulate mature-form microRNA expression. In contrast, miR-143/145 [48,49] and the miR-15a/16-1 [50] cluster are suppressed by chromosome deletion in leukemia. Genetic mutations or translocations also lead to the downregulation of miR-15a/16-1 in chronic lymphocytic leukemia (CLL) or miR-17/92 cluster expression in T-cell acute lymphoblastic leukemia [51,52] (Figure 2A). In addition to genomic translocation and mutation regulation of microRNA clusters, transcription factors (TFs) act on microRNA promoters and collaborate with RNA polymerase II (Pol II) to control microRNA cluster expression in cell-, tissue- or disease-specific manners. The aberrant expression of cancer specific transcription factors and mutation on microRNA cluster promoters contribute to cancer-specific microRNA cluster expression [53,54]. For example, MYC proto-oncogene controls miR-17/92 cluster expression [55] (Figure 2B). Epigenetic modifications are another important regulation mechanism for endogenous gene expression. *De novo* DNA methyltransferase (DNMT) 3A and 3B transfer adds a methyl group to the C5 positions of cytosine in CpG dinucleotides [56]. About 70% of gene promoters have a CpG island near the transcription factor start site [57,58]. DNA hypermethylation on the promoter CpG island leads to the recruitment of PRC complex repressors, which modifies specific histone methylation (H3K27me3) [59,60]. Eventually, HP1 joins the catalytic protein complex and converts euchromatin into heterochromatin [61,62]. In oral cancer, hypermethylation occurs on the miR-379/656 cluster promoter, where the genomic imprint for the non-coding RNA Meg3 differential methylation region (Meg3-DMR) also inhibits down-stream noncoding RNA and microRNA cluster expression [8,63] (Figure 2C). The abundance of single microRNA targets, like core transcription factors or key metabolic enzymes, can activate oncogenic signaling or change the intracellular metabolite balance to promote cancer progression [64,65]. Compare with single microRNA regulation, there are few studies focus on the regulation of microRNA clusters in cancer. Cluster microRNAs are evolution-conservation and usually coordinately expression of high homologue microRNAs which are usually belonged the same microRNA family. The coordinately expression of cluster microRNAs can not only accumulate contents of microRNAs but also the differential expression of microRNA targets. Minor regulation of the microRNA cluster has a huge impact on cancer tumorigenesis.

## 4. microRNA in Cancer

MicroRNAs was first discovered in *Caenorhabditis elegans* in 1993 [66,67]. Aberrant microRNA expression has been found in many diseases like cancer [68,69], neuron degeneration diseases [70], and developmental disabilities [71]. Cancer cells harbor various chromosome instabilities and mutations [72,73] which prompt the development of aberrant transcriptomes in both coding and non-coding genes. Aberrant microRNAs can be signaling transduction brokers between initiation genetic or epigenetic events and down-stream effector genes such as enzymes, signaling molecules, and traffic proteins for maintaining intracellular functions and intercellular commutations. Competing endogenous RNA (ceRNA) is a mechanism that RNA regulates other RNA transcripts by competing for shared microRNAs and microRNA binding elements [74,75,76,77] and include various products from transcriptome, such as protein coding genes, pseudogenes [78], long noncoding RNAs (lncRNAs) [79], and circular RNAs (circRNAs) [80,81]. Up- or down-regulation of one target may change the expression of other cognate targets by sequestering or releasing their shared microRNA molecules. Dysregulation of ceRNA network is important in cancer and other diseases [82,83,84]. MicroRNA families harbor seeding region sequence homologue, which is critical in regulation of ceRNA [85,86]. Some microRNA clusters are comprised of family microRNAs [87] and coordinate-expression [88] which hint microRNA clusters are important in ceRNA regulation. In ceRNA theory, microRNAs could act as hub molecules that promote cross-talk between different signaling pathways or biological functions. In cancer cells, aberrant microRNA molecules can be divided into master or driver molecules that can promote oncogenic cascades from a small signaling initiator toward a completely out of control situation. Differentially expressed microRNAs can also be defined as oncogenic microRNAs (OncomiRs) or tumor suppressor microRNAs (TSmiRs), based on the roles of microRNA regulation genes [89]. MicroRNAs that inhibit the expression of oncogenes, cell proliferation genes, or cancer progression genes can be called tumor suppressor microRNAs (Figure 1) [90,91,92]. In contrast to TSmiRs, microRNAs that target tumor suppressor genes or gene function while maintaining their epithelial status, like tight junction proteins on cell–cell hinge proteins, are called oncogenic microRNAs [93,94]. However, each type of microRNA can have different functions in different cell types [95].

## 5. microRNA in Cancer Microenvironment

Cancer cells are energy-hungry cells which demand endless nutrition for cell growth. Cancer cells refit surrounding normal cells into cancer-supporting fertile soil through secretion proteins such as cytokines, chemokines, and growth factors or exosomal microRNAs. Secretion proteins are paracrine ligands which bind directly to receptors on the surrounding cell surface and promote cell reorganization, migration, and proliferation. Tumor microenvironment comprises a mass of heterogeneous cell types, including, pericytes [96], endothelial cells [97], tumor-associated macrophages (TAMs) [98], and cancer-associated fibroblasts (CAFs) [99] all of which surround tumor cells as the core and cross-talk among surrounding normal cells. Angiogenesis is an essential process to provide oxygen and nutrient levels for tumor growth and metastasis [100]. During angiogenesis, blood vessels and stromal components, such as pericytes and endothelial cells, produces pro- and antiangiogenic factors and conduct many signaling pathways that allow vascular remodeling, neovascularization and communication with tumor cells. Vascular endothelial growth factor (VEGF) or basic fibroblast growth factor (bFGF) can stimulate pericytes and vascular endothelial cells reorganization into angiogenic vessels which can support nutrition and demand energy for cancer cell growth [101]. Unlike secretion proteins which are usually unstable in the serum and cancer microenvironments. Angiogenic genes may be regulated at the post-transcriptional level by microRNAs [100]. Exosomal microRNAs which are embedded in the small extracellular vesicle (sEV) and shaded by lipid bilayer have high stability and can act as paracrine or endocrine in the receipt cells through membrane-fusion. Exosomal microRNAs play critical role in interaction of tumor cells and tumor microenvironment and thereby modulates tumor progression and development [102]. Involvements of microRNAs in tumor cells, pericytes and endothelial cells during angiogenesis has been described in two comprehensive reviews by Salinas-Vera et al. [103] and Orso et al. [104].

Tumor-associated macrophages (TAMs) are the most abundant immune cells in the tumor microenvironment [105]. MicroRNAs notably influence the phenotype of TAMs through various targets and signal pathways during cancer progression and functions as important regulators in macrophage differentiation, functional polarization, and cellular crosstalk, which has been clearly described by Chen et al. [106]. Especially, microRNAs can be transported between tumor cells and macrophages via microvesicles and exosomes and facilitate crosstalk between tumor cells and macrophages, which is essential for tumor microenvironment formation and tumor progression [106].

Cancer-associated fibroblasts (CAFs) are a key component of the tumor microenvironment with diverse functions, including matrix deposition and remodeling, extensive reciprocal signaling interactions with cancer cells and crosstalk with other stromal cells by secreting various pro-inflammatory factors [107]. Changes of microRNAs expression in CAFs can be induced both by cancer cells and other stromal cells, which result from through direct interaction or by secreted paracrine factors or even by secreted microRNAs. MicroRNAs dysregulation contributes to CAFs phenotype and functions and assists their cancer promotion ability. CAFs interact with cancer cells by secreted microRNAs packaged in extracellular vesicles and exosomes and by directly releasing microRNAs into extracellular fluid. Wang Z. et al. provide the latest research about the relevance of microRNAs in the interaction between cancer cells and the CAFs. MicroRNAs from CAFs modulated cancer cells in various aspects, including cancer progression, metastasis, cancer metabolism, stemness and drug sensitivity [108].

MicroRNAs can circulate between various types of cells and regulate biologic effects, including supportive, inhibitory for tumor growth or dissemination. MicroRNAs can be released from cancer cells or microenvironment cells in various forms of vesicles or as “free” molecules secreted by active mechanisms [109]. Active participation of microRNAs in cancer cells and tumor microenvironment can be as therapeutic response predictors or therapeutic targets. By using sensitive detection technologies, microRNAs in body fluids or blood from patients with cancer can be detected and predicted a distinct microRNA expression profile [110]. MicroRNA is a critical mediator among tumor cells and tumor microenvironment and targeting microRNAs can be as promising therapeutic application.

## 6. Head and Neck Squamous Cell Carcinoma (HNSCC)

Head and neck squamous cell carcinomas (HNSCCs) represent the top lethal cancer type in the world. Although there have been great improvements in cancer management and treatment strategies in recent decades, which have extended the life quality and lifespan expectancy of most cancer patients. The HNSCC patient survival time or 5-year conditional survival time didn’t have a great improvement in the twentieth century [111]. The new target therapy, advanced radiotherapy, immune-checkpoint inhibitors treatment, and improvement surgeon skills could improve the HNSCC patient survival time [112]. HNSCCs exhibit a gender difference, with the male to female incidence ratio being 2.7:1. As for the mortality rate, males are again the most affected, with the ratio being 3.8:1. HNSCCs still cause close to 3000 deaths in Taiwan and 10,000 deaths in the United States per year [113]. Ninety percent of HNSCC cases originate in the oral cavity and are considered oral to squamous cell carcinomas. HNSCCs can be considered locoregional diseases; however, distant metastasis and local recurrence are major determinant factors in treatment management and cancer prognosis [114]. Cancers, including HNSCCs, are regarded as multifactorial diseases caused by various genetic or epigenetic modifications that induce the silencing of tumor suppressor genes, the activation of oncogenes, the escape of host immune detection and responses, and commutation with external microenvironments [115]. The major risk factors for the development of HNSCCs are alcohol consumption, habitual tobacco use, and betel nut chewing, as is common in southeast Asia. DNA damage by cigarette carcinogens such as tobacco-specific nitrosamines or arecoline from betel chewing are the major carcinogens responsible for HNSCC initiation [116,117]. Globally, smoking is the most preventable single carcinogen which cause of head and neck cancer with the highest users in the WHO European region (75.3%). Alcohol drinking is the second most common preventable factor which cause of HNSCC [118]. Chronic alcohol consumption and tobacco act synergistically in the development of head and neck cancer, while recent human papillomavirus (HPV) infection operates as an independent risk factor and is considered to have a large role in the development of oropharyngeal carcinogenesis [119,120,121]. Changes in tumorigenesis components including coding and non-coding genes modulate every step of cancer progression from tumorigenesis to angiogenesis, recurrence, and distant metastasis formation [112].

## 7. microRNA Deregulation in HNSCC Carcinogenesis

Abnormal microRNA expression has been reported during head and neck squamous carcinoma cancer (HNSCC) tumorigenesis and progression. Most clinical, experimental, and mechanistic studies of microRNA have focused on global patterns [122,123] of microRNA signatures which are not genetically linked or single microRNA functions in HNSCC. The functions of single oncogenic microRNAs (OncomiRs) or tumor suppressor microRNAs (TSmiR) are summarized in Table 2. miR-450a and miR-455 serve as OncomiRs which contribute tumor progression by targeting TMEM182 and UBE2B, respectively [124,125]. miR-31 has multiple targets, such as SIRT3, FIH, and ARID1A, and also can be cancer biomarkers in the plasma or serum [64,126,127,128]. miR-155 inhibits tumor suppressors and cell division cycle 73 (CDC73) in OSCC [129], and the up-regulation of miR-155 promotes OSCC cervical lymph node metastasis and is associated with a poor overall survival rate in patients [130,131,132]. In contrast to OncomiRs, miR-376c suppresses the expression of the transcription factor RUNX2 and inhibits the growth and metastasis of OSCC cells [63]. miR-379 and miR-30a regulate the expression of DNA methyltransferase 3B (DNMT3B) and the OSCC epigenome, preventing cancer progression [133]. miR-486 targets the cell membrane tyrosine kinase receptor DDR1 and prevents OSCC cell growth [134]. miR-99a governs insulin-like growth factor I receptor signaling [135]. However, most current microRNA studies focus on the association between one-microRNA and one target gene function in cancer. Although single microRNA may be sufficient in tumor biology, the cluster microRNAs have several coordinately regulated microRNAs which can accumulate contents of family microRNAs and aberrant expression of target genes from individual microRNAs in the cluster. Dysregulation microRNA cluster is an energy-saving and broad-impact procedure than single microRNA in cancer development or progression.

## 8. microRNA Cluster Deregulation in HNSCC

Maintaining life is an energy-consuming process. Cancer cells usually use all of their energy to grow or resist the external environment. Cluster microRNAs usually belong to a single microRNA family, which can enhance their regulation efficiency, and their coordinated regulation by a single genetic or epigenetic effect can save cellular energy for tumorigenesis and cancer progression. Regulation of microRNA cluster is an energy-saving and robust way to change cancer cell fate. Our previous studies focused on the expression, regulation mechanisms, and subsequent functions of aberrant microRNA clusters in HNSCC cancer tumorigenesis and progression [8,188]. DLK1-MEG3 imprinting locus has a huge miR-379/656 cluster which is epigenetic silenced in Taiwan OSCC patents by betel nut exposure. Arecoline, an active alkaloid from betel nut, cause DNA hypermethylation on differentially methylated region (DMR) on DLK1-MEG3 locus and silence whole imprinting locus expressions [8,63,133]. The miR-17/92 cluster has bi-functional role in OSCC growth and migration. The miR-17/92 cluster promotes OSCC growth by targeting FOXP1 [196] but it also suppresses cell migration and invasion by targeting integrin β8 [197]. Activin A is growth promoting autocrine [65]. Down regulation of miR-143/145 cluster synergistic promotes Activin A expression and serves as a poor prognostic marker in oral cancer patients [198]. Another tumor-suppressive microRNA cluster, microRNA-23b/27b, regulates oncogenic MET receptor tyrosine kinase expression and cell migration in OSCC cells [199]. In HNSCC, oncogenic miR-106b/25 cluster including miR-106b, miR-93 and miR-25 [200,201,202] affect cell proliferation [200] via targeting tumor suppressor p21/CDKN1A pathway [203] Upregulation of the miR-371/372/373 microRNA cluster enhances oral cancer oncogenicity and drug resistance [204]. Furthermore, the miR-503/450b cluster on the chromosome X promotes oral cancer progression and this cluster is controlled by inflammation cytokine interleukin 8 and signal transducer and activator of transcription 5 (STAT5) pathway [124,188]. Aberrant microRNA in HNSCC is listed in Table 2.

## 9. Translational Application of the miR-503/450b Cluster

Exosomes are small cell-membrane-like extracellular vesicles that contain cellular biomolecules such as proteins, mRNAs, non-coding RNAs, and microRNAs for intercellular communication from donor to recipient cells via target cell membrane fusion [205,206,207]. The simplest way for exosome cargo loading to occur is by simple diffusion, and the high abundance of biomolecules in donor cells can allow cargo to diffuse into exosomes by a concentration gradient [208]. The lipid-bilayer structure of exosomes can protect the inner cargo, preventing degradation by serum protease or ribonuclease and creating an extremely high level of stability for cargo molecules [209]. The high stability of exosomal biomolecules makes them suitable for liquid biopsy in clinical diagnosis [210]. microRNAs on the miR-503/450b cluster are the top-ranking expression microRNAs in OSCC tumors [8,211,212,213,214]. Furthermore, these microRNAs can be found in exosomes from T cells [215], dendritic cells [215], endothelial cells [216], and serum [217,218] in non-cancer diseases, such as early onset type 1 diabetes [219] and ischemia [220]. Taken together, these studies reveal the potential roles of the miR-503/450b cluster in immune and cancer microenvironment regulation, and microRNAs within this cluster are stable in the serum and circulation system so they can be delivered to recipient cells or can be detected by current detection technologies. Recently, the miR-503-containing exosome has been used in endothelial-derived breast cancer antitumor neoadjuvant chemotherapy [221]. Thus, microRNAs within the miR-503/450b cluster not only have diagnosis or prognosis functions, but also have potential for use in future cancer therapies.

## 10. Summary and Perspectives

According to previous microRNA studies, the microRNA cluster is a missing link in cancer research fields. Regulation of the microRNA cluster is an energy-efficient procedure involving tumorigenesis, cell growth, metastasis, and cancer progression. A tiny change in the microRNA cluster can tremendously alter cell functions. Usually, microRNAs in a cluster belong to the same family with high sequence homology. Thus, they can increase the regulation intensity in cancer cells or can be easily amplified in liquid biopsy. Previously, small RNA molecules could only work as cancer biomarkers and were detected by liquid biopsy. Improvements in nucleotide chemistry and modification technology have made RNAs much more stable in modern pharmaceuticals [222,223,224]. Inclisiran is the first U.S. Food and Drug Administration (FDA)-approved small interfering RNA drug that can inhibit PCSK9 translation to treat atherosclerotic cardiovascular disease (ASCVD) including hypercholesterolemia or mixed dyslipidemia [225,226,227,228]. The distribution of extracellular microRNA by antisense oligonucleotides may prevent intercellular communication among cancer microenvironments and cancer progression. microRNA research may have exciting prospects for cancer diagnostics, prognostics, and therapeutics. In the past few decades, there has been a tremendous change from the profiling of altered microRNA expression in cancers to clinical trials with microRNAs as therapy candidates. In the near future, we hope that microRNA replacement and inhibition therapies will enter pre-clinical and clinical pilot studies and potentially be used as personalized medicines.

## Figures and Tables

**Table 1:** The physiological functions of microRNA clusters.

**miRNA Function**	1/133a	106a/363	106b/25	143/145	15a/16-1	17/92	181a/181b	183/182	193/365a	199a/241	200b/429	206/133b/	212/132	221/222	23a/24-2	27a/27b	29b/29a	301b/130b	302b/367	34b/34c	371a/373	379/656	493/136	424/450b	508/513a	512/519
Differentiation and development			◎		◎	◎						◎	◎		◎				◎	◎		◎	◎	◎		◎
Secondary messengers	◎	◎	◎		◎	◎		◎					◎	◎	◎											
microRNA and stress			◎	◎		◎									◎					◎				◎		
Immunity						◎	◎	◎							◎											
Feedback loops						◎					◎				◎											
Cell junctions and adhesions						◎									◎											
Mammalian reproductions			◎			◎													◎	◎	◎					
Cardiac development and diseases	◎		◎	◎		◎																				
Cellular reprogramming		◎				◎													◎		◎					
Cell to cell communication						◎																◎	◎			◎
DNA repair						◎												◎							◎	
Viral infection		◎				◎																				
Nervous system regulation							◎						◎				◎					◎				
Metabolism								◎	◎						◎				◎			◎		◎		
Organellar biogenesis and function			◎							◎					◎	◎			◎							
Mesenchymal to epithelial transition			◎																◎					◎		

**Table 2:** Aberrant microRNA in HNSCC.

microRNA	Function	Targets	Effects on Tumor	Reference
miR-1	TSmiR	TAGLN2, PNP	Proliferation − Apoptosis +	[136]
miR-1	TSmiR	TAGLN2	Proliferation − Apoptosis + Cell cycle arrest +	[137]
miR-1	TSmiR	EGFR, c-Met	Proliferation − Migration − Invasion −	[138]
miR-1	TSmiR	Slug	Migration − Invasion − Stemness −	[139]
miR-1	TSmiR	ET-1	Angiogenesis −	[140]
miR-7	TSmiR	IGF1R	Proliferation − Apoptosis + Cell cycle arrest +	[141]
miR-9	TSmiR	PTEN	Proliferation −	[142]
		CXCR4	Proliferation − Apoptosis + Cell cycle arrest + Invasion −	[143]
miR-100	TSmiR	IGF1R, mTOR	Proliferation − Apoptosis + Migration −	[144]
		Akt1	Proliferation − Apoptosis + Migration −	[145]
miR-101	TSmiR	EZH2, rap1GAP	Proliferation − Invasion −	[146]
		EZH2	Migration − Invasion − EMT −	[147]
miR-101	TSmiR	ITGA3	Migration − Invasion − Angiogenesis −	[148]
miR-101-3p	TSmiR	Pim-1	Proliferation − Apoptosis + Invasion −	[149]
miR-104-5p	TSmiR	PAK4	Proliferation − Apoptosis + Cell cycle arrest +	[150]
miR-104-5p	TSmiR	Survivin	Proliferation − Apoptosis + Invasion −	[151]
miR-10b	TSmiR	-	Proliferation − Cell cycle arrest +	[152]
miR-10b	OncomiR	-	Migration + Invasion +	[153]
miR-10b	OncomiR	-	Migration + Invasion +	[154]
miR-1-3p	TSmiR	DKK1	Proliferation − Apoptosis + Cell cycle arrest + Migration − Invasion −	[155]
miR-125b	TSmiR	PRXL2A	Oxidative stress + Drug sensitivity +	[156]
miR-138	TSmiR	GNAI2	Proliferation − Apoptosis +	[157]
miR-140-5p	TSmiR	ADAM10	Migration − Invasion −	[158]
miR-140-5p	TSmiR	LAMC1, HDAC7, PAX6	Migration − Invasion −	[159]
miR-146a	TSmiR	-	Proliferation − Migration −	[132]
miR-155	TSmiR	-	Proliferation − Migration −	[132]
miR-155	OncomiR	-	Association with tobacco chewing	[160]
miR-155	OncomiR	CDC73	Proliferation + Migration +	[129,130,131]
miR-181a	TSmiR	K-ras	Proliferation −	[161]
miR-181a	TSmiR	MAP2K1, MAPK1, Snai2	Proliferation − Migration − Invasion −	[162]
miR-181a	TSmiR	Twist1	Drug sensitivity + EMT − Metastasis −	[163]
miR-181a/b	OncomiR	-	Migration − Invasion −	[164]
miR-183	OncomiR	-	Prognostic biomarker	[165]
miR-196a	OncomiR	AXIN	Proliferation − Cell cycle arrest +	[152]
miR-21	OncomiR	-	Prognostic biomarker	[165]
miR-21	OncomiR	TPM1, PTEN, CDK2AP1, HIF-1A, HIF-2A	Association with tumor progression	[166]
miR-203	OncomiR	-	As diagnostic marker of metastasis	[167]
miR-203	TSmiR	-	Invasion − EMT −	[168]
miR-204	TSmiR	BRD4	Proliferation − Apoptosis + Cell cycle arrest +	[169]
	TSmiR	Sox4, Slug	Stemness − EMT − Metastasis −	[170]
	TSmiR	CDC2	Invasion − Metastasis −	[171]
miR-204-5p	TSmiR	CXCR4	Proliferation − Cell cycle arrest + Migration − Invasion −	[172]
miR-205	OncomiR	-	As diagnostic marker of metastasis	[167]
miR-206	TSmiR	EGFR, c-Met	Proliferation − Migration − Invasion −	[138]
miR-221	OncomiR	p27, p57	Proliferation +	[173]
		PTEN	Proliferation + Invasion + Apoptosis −	[174]
miR-222	OncomiR	p27, p57	Proliferation +	[173]
		PTEN	Proliferation + Invasion + Apoptosis −	[174]
miR-222	TSmiR	PUMA	Proliferation − Invasion − Drug sensitivity +	[175]
miR-222	TSmiR	MMP1, SOD2	Invasion −	[176]
miR-223	OncomiR	-	Proliferation − Apoptosis +	[177]
miR-223	OncomiR	FBXW7	Proliferation + Migration +	[178]
miR-24	OncomiR	DND1	Proliferation + Apoptosis −	[179]
miR-25	TSmiR	NEDD9	Proliferation − Apoptosis +	[180]
miR-26a	TSmiR	DNMT3B	Proliferation − Cell cycle arrest + Apoptosis +	[181]
miR-26a/b	TSmiR	PAK1	Cell cycle arrest + Migration − Invasion − Apoptosis + Glycolysis −	[182]
miR-26a/b	TSmiR	TMEM184B	Migration − Invasion −	[183]
miR-31	OncomiR	-	As diagnostic biomarker	[126,127]
		SIRT3	Migration + Invasion +	[64]
		FIH	Migration + Invasion +	[128]
		ARID1A	Oncogenesis + Stemness +	[184]
miR-34a	TSmiR	MDM4, SIRT1	Downregulated in tumors	[185]
miR-34a	TSmiR	BCL-2	Migration − Invasion −	[186]
miR-372	OncomiR	ZBTB7A	Drug sensitivity −	[187]
miR-376c	TSmiR	RUNX2	Migration − Invasion − Lymphatic metastasis −	[63]
miR-379	TSmiR	DNMT3B	Proliferation +	[133]
miR-424	OncomiR	SOCS2	Migration + Invasion +	[188]
miR-450a	OncomiR	TMEM182	Migration + Invasion +	[124]
miR-455	OncomiR	UBE2B	Proliferation +	[125]
miR-486	TSmiR	DDR1	Proliferation − Apoptosis +	[134]
miR-503	OncomiR	Smad7	Proliferation + Migration + Invasion +	[189]
miR-494	TSmiR	HOXA10	Proliferation −	[190]
miR-494-3p	OncomiR	Sox7	Proliferation + Migration + Invasion +	[191]
miR-99	TSmiR	AKT1	Proliferation − Apoptosis + Migration −	[145]
miR-99-3b	TSmiR	GSK3b	Proliferation −	[192]
miR-99a	TSmiR	mTOR	Proliferation −	[193]
		MTMR3	Migration − Invasion −	[194]
miR-99a	TSmiR	IGF1R	Migration − Invasion −	[135]
let-7b	TSmiR	Dicer	Proliferation −	[195]

Both (−) and (+) mean microRNAs show inhibitory or stimulative effect on head and neck cancer, respectively. Abbreviations: ADAM10, ADAM metallopeptidase domain 10; AKT1, AKT serine/threonine kinase 1; ARID1A, AT-rich Interaction domain 1A; AXIN, Axis inhibition protein 2; BCL-2, B-cell lymphoma 2; BRD4, Bromodomain-containing protein 4; CDC42/73, Cell division cycle 42/73; CDK2AP1, Cyclin-dependent kinase 2-associated protein 1; c-MET, Hepatocyte growth factor receptor; CXCR4, C-X-C chemokine receptor type 4; DDR1, Discoidin domain receptor-1; DKK1, Dickkopf wnt signaling pathway inhibitor 1; DND1, RNA-binding protein dead end 1; DNMT3B, DNA (cytosine-5-)-methyltransferase 3 beta; EGFR, Epidermal growth factor receptor; ET-1, Endothelin-1; EZH2, Enhancer of zeste 2 polycomb repressive complex 2; FBXW7, F-Box and wd repeat domain containing 7; FIH, Factor-inhibiting hypoxia-inducible factor; GNAI2, G protein subunit alpha I2; GSK3b, Glycogen synthase kinase 3 beta; HDAC7, Histone deacetylase 7; HIF-1/2A, Hypoxia inducible factor 1/2 alpha; HOXA10, Homeobox A10; IGF1R, Insulin-like growth factor 1 receptor; ITGA3, Integrin subunit alpha 3; K-ras, KRAS proto-oncogene; LAMC1, Laminin subunit gamma 1; MAP2K1, Mitogen-activated protein kinase kinase 1; MDM4, Mouse double minute 4 homolog; MMP1, Matrix metallopeptidase 1; MTMR3, Myotubularin-related protein 3; mTOR, Mammalian target of rapamycin; NEDD9, Neural precursor cell expressed, developmentally down-regulated 9; p27, Cyclin dependent kinase inhibitor 1b; p57, Cyclin dependent kinase inhibitor 1c; PAK1/4, p21-activated kinase 1/4; PAX6, Paired type homeobox 6; Pim-1, Serine/threonine-protein kinase Pim-1; PNP, Purine nucleoside phosphorylase; PRXL2A, Peroxiredoxin like 2a; PTEN, Phosphatase and tensin homolog; PUMA, BCL2 binding component 3; rap1GAP, RAP1 GTPase activating protein; RUNX2, RUNX Family transcription factor 2; SIRT1, Sirtuin 1; SIRT3, Silent information regulator 3; Slug, SNAI2, snail family transcriptional repressor; Smad7, SMAD family member 7; Snai2, Snail family transcriptional repressor 2; SOCS2, Suppressor of cytokine signaling 2; SOD2, Superoxide dismutase 2; Sox4/7, SRY-related HMG-box 4/7; Survivin, BIRC5, baculoviral IAP repeat containing 5; TAGLN2, Transgelin 2; TMEM182/184B, Transmembrane protein 182/184B; TPM1, Tropomyosin 1; Twist1, Twist family BHLH transcription factor 1; UBE2B, Ubiquitin conjugating enzyme E2 B; ZBTB7A, Zinc finger and BTB domain containing 7A.
